# Is invasion a necessary step for metastases in breast cancer?

**DOI:** 10.1007/s10549-017-4644-3

**Published:** 2018-01-20

**Authors:** Steven A. Narod, Victoria Sopik

**Affiliations:** 1 Women’s College Research Institute, Women’s College Hospital, 76 Grenville Street, Toronto, M5S 1B2 Canada; 2 Dalla Lana School of Public Health, University of Toronto, Toronto, Canada; 3 Institute of Medical Science, University of Toronto, Toronto, Canada

**Keywords:** Breast cancer, DCIS, Invasion, Metastasis, Death

## Abstract

**Purpose:**

To review the empirical evidence to support the conventional (sequential) model of breast cancer progression, which is based on the paradigm that cancer passes through several stages, including an in situ stage prior to an invasive stage, and thereafter (in some cases) disseminates to the lymph nodes and distant organs.

**Methods:**

We review the cancer literature of the last 50 years which relates to the prevention of invasive breast cancer (through radiotherapy or surgery) and reductions in the mortality for breast cancer.

**Results:**

For both invasive cancers and DCIS, the literature indicates that prevention of in-breast invasive recurrences does not prevent death from breast cancer. Moreover, the presence of residual cancer cells in the breast after breast-conserving surgery does not compromise the cure rate.

**Conclusion:**

We propose an alternate (parallel) model of breast cancer wherein there is a small pool of cancer stem cells which have metastatic potential from their inception and which disseminate synchronously through several routes—to the breast stroma, to the lymph nodes and to distant organs. Cancer cells which disseminate to the breast give rise to cells which make up the bulk of the tumour mass but these are not the source of the distant metastases.

## Part 1: The systemic nature of breast cancer

Among the milestones reached in the 20th century regarding the treatment of women with early stage breast cancer was the discovery that mortality was comparable for patients treated with mastectomy and with breast-conserving surgery (lumpectomy). Fisher [1] and Veronesi [2] challenged the iconic view put forward by Halsted nearly a century before [3] that breast cancer arose at a single location and spread locally within the breast and then to the regional lymph nodes before it became more widely disseminated. The new ‘systemic’ model meant that, in some cases, the breast cancer will have spread beyond the breast before diagnosis, and in these cases, it was necessary to provide systemic treatment if all cancer cells were to be eliminated. The finding of equivalence of the two surgical approaches for treating women with small cancers was lauded as a breakthrough by patient advocacy groups and their support hastened the acceptance of breast-conserving surgery in the 80s and 90s [4–6]. By changing their practice accordingly, most surgeons tacitly endorsed Fisher’s new ‘systemic’ model of breast cancer progression—but few explored the theoretical implications of the salient observations. It has been known for some time, that about one-third of women with breast cancer, following lumpectomy, experience an invasive recurrence in the same breast [1, 2]. As expected, the risk of ipsilateral invasive recurrence is much lower for women treated with mastectomy than for women treated with lumpectomy [1, 2]. Furthermore, women who experience an in-breast invasive recurrence following lumpectomy are at much greater risk for death from breast cancer than are women who do not experience a recurrence [7]. However, neither in the American trial nor in the Italian trial was it found that the prevention of a local invasive recurrence through mastectomy was associated with a decline in breast cancer mortality [8, 9]. Elsewhere, trials of radiotherapy post-lumpectomy showed similar results, namely that radiotherapy was highly effective in preventing local recurrence, but the preventive effect of radiotherapy on breast cancer mortality was smaller than the preventive effect on local invasive recurrence [10, 11]. These observations, which continue to puzzle us today, have profound implications, which we explore here.

## Part 2: The SEER study: mortality after DCIS

**Fig. 1:**
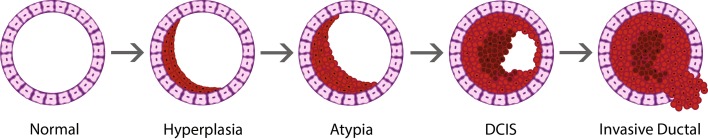
Cross section of a breast duct showing progression of invasive ductal carcinoma. Reproduced from [14]

Recently, in a study based on the US SEER Cancer Registry, we reported rates of local recurrence and breast cancer mortality in patients with ductal carcinoma in situ (DCIS) [12]. We will review some background material regarding DCIS before returning to the study itself. DCIS refers to the presence of neoplastic breast cells within a breast ductule or lobule in the absence of neoplastic cells that breach the basement membrane (Fig. 1) [13, 14].

It has been inferred from the observation that small clusters of cancer cells often lie entirely within a duct (terminal duct-lobular units) and from studies of molecular markers of cell lineage, that most invasive ductal-type breast cancers originate within the epithelial lining of the breast duct [15, 16]. Areas of DCIS are often found in contiguity with areas of invasive cancer [17]. It has been proposed that breast cancer resembles colon cancer and other carcinomas in that the first-born cancer cells are adjacent to the basement membrane (and associated myoepithelial cell layer in the case of breast cancer) and later, after invasion through the basement membrane, the cancer enters a higher stage wherein malignant cells are contiguous with the stroma and connective tissues of the breast [18].

On a cellular level, DCIS cells look like invasive cancer cells [19]. Despite this, the majority of scholars are of the opinion that, because of its position vis a vis the basement membrane, DCIS is a *precursor* of invasive cancer, i.e. DCIS is not frankly malignant in its own right (and is not life-threatening), but some cases of DCIS can ‘progress’ to invasive breast cancer [20]. In this scenario, the invasive in-breast recurrence post-DCIS is considered a primary invasive cancer. Some suggest that the name ‘DCIS’ should be changed to reflect the benign nature of the condition [21]. A minority of investigators consider DCIS to be a bonafide cancer in its own right, with the potential to metastasize in the absence of invasion through the basement membrane and which, on its own, poses a small but significant threat to life [12].

In our SEER-based study, among patients with DCIS, the risk of invasive local recurrence 10 years after treatment was 1.3% for women with mastectomy, was 2.5% for women with lumpectomy and radiation, and was 4.9% for women with lumpectomy without radiation [12]. The risk of death from breast cancer after treatment was 1.3% for women with mastectomy, was 0.8% for women with lumpectomy and radiation, and was 0.9% for women with lumpectomy without radiation. That is, among DCIS patients, mastectomy reduced the risk of local recurrence by 75%, but did not reduce the risk of dying of breast cancer. Likewise, radiotherapy after DCIS reduces the risk of local recurrence by 50% but did not reduce the risk of dying of breast cancer. In the largest study of its kind prior to our study (EBCTG 2010) [22], 3729 women with DCIS were treated with breast-conserving surgery and then were randomized to radiotherapy or to no radiotherapy. Radiotherapy reduced the risk of ipsilateral invasive cancer by more than one-half (204 cases versus 92 cases) but was associated with a small but non-significant *increase* in breast cancer mortality (44 deaths versus 52 deaths; HR 1.22). The authors do not conclude that preventing local invasive recurrence does not reduce mortality; rather, they dismiss this incongruity somewhat cryptically: “the differences are not significant, chance seems to be a likely explanation for them”. In the SEER study [12], approximately one-half of the DCIS patients who died of breast cancer did not have an invasive cancer recorded prior to their death from breast cancer. This observation supports our position that DCIS is a cancer in its own right. Similar findings were also seen in a recent study of 9799 DCIS cases from The Netherlands [23]. In that study, the probability of developing an ipsilateral invasive breast cancer was much higher for women treated with lumpectomy (308 of 2558; 12.0%) than for women treated with mastectomy (68 of 4667; 1.5%). However, the absolute risks of breast cancer mortality at 10 years for women with lumpectomy (2.7%; 95% CI 2.1–3.4) and mastectomy (2.6%; 95% CI 2.1–3.1) were similar. Furthermore, of the 284 women who died of breast cancer, only 43% experienced a prior invasive breast cancer (ipsilateral or contralateral).

A large case–control study from Sweden also came to similar conclusions [24]. In that study, 96 women diagnosed with primary DCIS between 1992 and 2012 who later died of breast cancer were identified and compared with a group of 318 controls (women diagnosed with primary DCIS who were alive at the time of death of the corresponding case). Treatment by mastectomy or the addition of radiotherapy after lumpectomy was not associated with a lower risk of subsequent death from breast cancer. The odds ratio for death from breast cancer for patients treated with lumpectomy plus radiotherapy (versus with lumpectomy alone) was 1.46 (95% CI 0.81–5.63) and that for patients treated with mastectomy (versus with lumpectomy alone) was 2.26 (95% CI 1.29–4.06). Furthermore, of the 96 cases, 15 developed distant metastases without a preceding ipsilateral or contralateral invasive breast cancer. The authors report that all medical records for cases and controls were retrieved and that the follow-up of subsequent breast events and causes of death was complete. The authors suggest that these results may indicate that some DCIS has an inherent potential for metastatic spread [24].

The appearance of a local invasive recurrence after DCIS is predictive of a large increase in the risk of dying of breast cancer; some will argue that this supports the notion that the invasive recurrence is the *bona fide* malignancy. For example, Wapnir et al. reported that after local invasive recurrence in a patient with DCIS, the mortality rate increases 7.1 times [25]. In our SEER study, invasive recurrence increased subsequent breast cancer mortality 18.1 times [12]. However, this does not necessarily imply that that the recurrence has metastatic potential—it could be a marker of aggressivity.^1^

Another criticism raised against our paper and the message that DCIS could be fatal in its own right was that pathology is an inexact science and that the 3% of women who succumbed to breast cancer probably had cancers with missed foci of micro-invasion, and hence, many invasive breast cancers were misclassified as DCIS [26]. In the SEER data set, there were 13,489 cases of DCIS with micro-invasion that we excluded from our original analysis. In SEER, the 10-year mortality rate from breast cancer was 2.8% for patients with DCIS with micro-invasion and was 1.4% for women with DCIS without micro-invasion [27]. If the mortality of DCIS patients was confined to the subgroup of patients with micro-invasion, then 50% of DCIS patients would have to have occult micro-invasion to generate these observed rates and the concept of DCIS being a discrete category would be challenged.

Others expressed the opinion that DCIS is highly heterogeneous and we cannot assume that all women with DCIS form a single category [28]. Perhaps, 3% of cases of DCIS are, in fact, cancer, but these few cases are outliers and the vast majority of cases of DCIS (97%) are not cancer. By the same logic, one could claim that more than 90% of stage I breast cancers are not cancer.

Support for the notion that breast cancer dissemination can occur in pre-invasive stages of tumour progression comes from the consistent finding of circulating tumour cells in the peripheral blood (or in the bone marrow) of patients with DCIS. In six studies (range 19–404 patients), between 13 and 25% of DCIS patients were found to have circulating tumour cells [29–34]. It has been proposed that such cells derive from an occult (micro)-invasive lesion within DCIS. However, there is no difference in the frequency of circulating tumour cells in patients with DCIS, compared to those with frank invasive breast cancer [29–31]. In one study, disseminated tumour cells were detected in the bone marrow of 25% of 24 patients with pure DCIS and in 20% of 56 patients with (non-metastatic) invasive cancer (*p* = 0.57) [29]. In another study, circulating tumour cells were detected in the peripheral blood of 18.7% of 48 patients with pure DCIS and in 18.8% of 404 patients with non-metastatic invasive breast cancer [30]. The clinical significance of detecting circulating tumour cells in patients with DCIS is still unclear, but raises the important point that cells may enter circulation prior to passing the basement membrane.

Several pathology studies have reported that a significant proportion of patients with a final diagnosis of pure DCIS have positive lymph nodes (about 3%) [35]. Nodal disease is postulated to indicate that occult invasion must be present in the primary tumour. Based on this assumption, the SEER classification automatically assigns cases of DCIS with lymph node metastasis to stage II (or stage III). However, this prejudges the importance of the observations—there is now consistent evidence that some women with confirmed DCIS will have positive lymph nodes, even when comprehensive tissue sectioning reveals no occult invasion [36, 37]; this observation is important and reflects a need to re-examine the association between non-invasive tumours and lymph node involvement.

A possible mechanism by which DCIS cells may enter circulation without breaching the basement membrane has been described for mucinous DCIS. In a study of 36 patients with mucinous DCIS, small vessels were identified within the lumen duct in 26 cases (72%) [38]. The authors propose an alternative pathway for invasion whereby intra-ductal mucin production promotes neovascularization [38], but this is a rare subtype of DCIS.

A further possibility for the induction of metastases in patients with DCIS may be the iatrogenic dissemination of tumour cells at the time of pre-operative biopsy. Incisional biopsy including the diagnostic core needle biopsy has been associated with seeding of tumour cells into the circulation and has been hypothesized to increase the risk of metastatic disease [39]. Seeding of the needle tract and mechanical displacement of cells into the lymphatic or vascular system has been documented in DCIS [40, 41], but the clinical implications are not known. Liebens et al. reviewed the clinical significance of malignant epithelial cell displacement after core needle biopsy in breast cancer patients [42]. Tumour cell displacement on surgical specimens occurred in 22% of the patients overall, with similar rates seen for DCIS and invasive breast cancer [40–42]. However, tumour cell displacement was seen less frequently as the interval between biopsy and surgical excision lengthened, suggesting that seeded cells do not survive displacement [41, 42]. No studies to date have documented a survival disadvantage with pre-operative biopsy [42].

From these observations, we conclude that the patterns of local recurrence and of mortality after DCIS and after primary invasive breast cancer are qualitatively similar and differ only by degree. Fisher and Veronesi pointed out that after primary invasive cancer, preventing local recurrence (by mastectomy versus lumpectomy) does not reduce mortality (if it did then we would recommend mastectomy) [7, 8]. The same holds true for DCIS. By extension, an in-breast invasive event following a diagnosis of DCIS might better be described as a local invasive recurrence than as a new primary cancer. However, what has been accepted for invasive cancer has not been accepted for DCIS.

To summarize: if an in-breast invasive recurrence observed after DCIS were a primary breast cancer, then preventing it should also prevent subsequent metastases (which could potentially be fatal), and if so, preventing the recurrence should prevent (at least some) deaths. However, preventing the invasive in-breast recurrence after DCIS did not result in fewer deaths—therefore, the recurrence is not a primary invasive cancer—therefore, the DCIS has inherent malignant potential. Our position here has not been widely accepted—most commentators continue to describe DCIS as a precursor lesion with little or no inherent malignant potential [43].

**Fig. 2:**
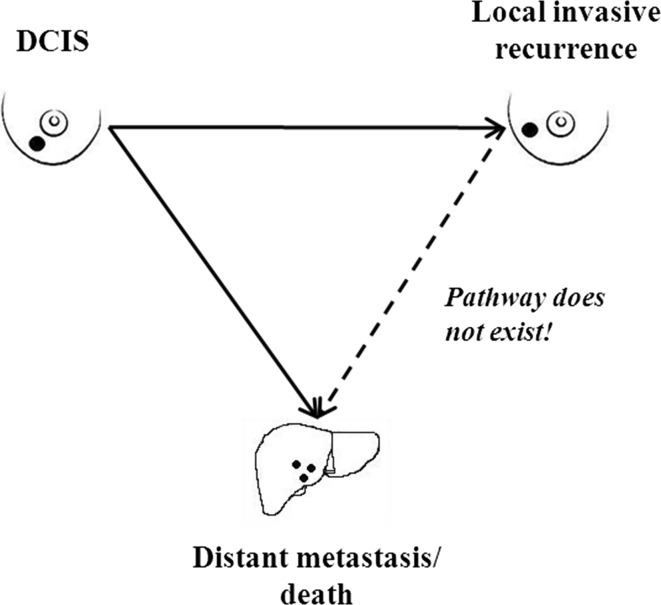
Proposed relationship between DCIS, local recurrence, and distant metastasis/death

If we are willing to accept that the DCIS is the primary malignancy and that an in-breast invasive event following a treated case of DCIS is a local recurrence, then the relationship between DCIS and death can be represented as in Fig. 2. There are two separate pathways; one leads from DCIS to local recurrence and one leads from DCIS to distant recurrence. There is no analogous pathway from local recurrence to distant recurrence—this leads us logically to accept that local recurrences do not have the potential to metastasize (again, if a local recurrence could metastasize, then a local recurrence would be a life-threatening event, but *preventing local recurrence after DCIS does not prevent death*; therefore, the local recurrence does not metastasize). The same logic holds true for local recurrences following primary invasive breast cancers.

## Part 3: The stage distributions of breast cancer in the clinic and in the population

**Fig. 3:**
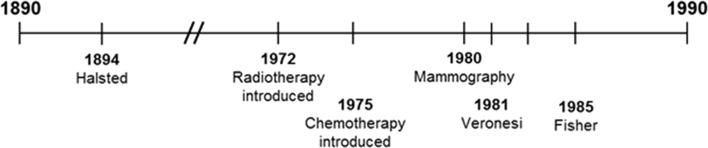
Historical timeline of breast cancer: 1890–2000 (see Box A: Historical timeline of breast cancer in Appendix)

Our current view of the natural history of breast cancer is the product of an incremental understanding of breast cancer illuminated over the last century, based on a chronological sequence of observations made about the timing of breast cancers and of cancer metastases (Fig. 3, Box A: Historical timeline of breast cancer in Appendix). The various states (and stages) of breast cancer have revealed themselves slowly as techniques of imaging, both at a gross level (through mammography) and at a microscopic level (through histology and immunohistochemistry) have been refined. Prior to the 20th century, breast cancer patients often presented with extensive local disease, which might involve infection and ulceration. Later, it was recognized that an asymptomatic mass detected on physical examination might be cancerous and that this suspicion could be confirmed by biopsy and histology [44]. This was the state of the art of breast cancer detection and diagnosis until late in the 20th century, when mammography screening became commonplace. In the late 1970s, small intra-ductal lesions were seen in pathology specimens with increasing frequency and these were described as DCIS [45]. At this time, DCIS was considered to be a special histologic subgroup of breast cancers rather than an early phase of breast cancer itself.

The relative numbers of women in the entire population with DCIS, with organ-confined breast cancer, and with disseminated breast cancer at a given time can be crudely estimated by the proportions of cancers diagnosed at various stages (Table 1). In the absence of screening, there are few cases of DCIS; 90% of cases are diagnosed at stages I–III and about 10% are diagnosed at stage IV [46]. For screen-detected breast cancers, the distribution shifts: 20% are DCIS, 75% are stages I–III, and 5% are stage IV [47]. At autopsy, most cases are in situ. We can speculate on the actual distribution of cancers in the entire underlying population of women (both detected and undetected) based on post-mortem pathology studies [48] or using new and highly sensitive screening modalities, such as MRI [49]. The likelihood of a cancer being detected, if present, depends on the size of the cancer, its palpability, the frequency of screening, and the sensitivity of the particular screening test. We can assume that every breast cancer passes through an undetectable phase at which time it is missed by our most sensitive screening tools. For this reason, the observed ratio of 1–3 for DCIS to invasive cancers in clinic patients is probably an underestimate of the true ratio.

**Table 1:** Relative stage distribution of breast cancers, by mode of ascertainment

Mode of ascertainment	Stage 0 (DCIS)	Stage I–III (%)	Stage IV
Clinical examination [47]	Few	90	10%
Mammography [47]	20%	75	5%
MRI [49]	33%	67	Low
Autopsy [48]	90%	10	0%

### Reprise

At this point in the essay, we have argued that the condition called DCIS came into prominence in the latter half of the 20th century as pathology techniques improved and as mammography screening identified increasing numbers of women with small, non-palpable asymptomatic breast cancers; that because of various issues of ascertainment, the actual number of cases of DCIS in the population at any given time is likely to exceed the number of cancers at a later stage, in contrast to the greater number of invasive versus non-invasive cancers in lesions removed by surgeons and classified by pathologists. We have shown that an invasive in-breast event following a case of DCIS that has been surgically removed is better characterized as local invasive recurrence rather than as a new primary cancer, and we propose that local recurrences following DCIS do not have the ability to metastasize. We now explore the implications of these hypotheses regarding primary invasive breast cancer.

## Part 4: Are primary invasive breast cancers and local invasive recurrences following DCIS distinct?

### A tale of three women: a thought experiment

**Fig. 4:**
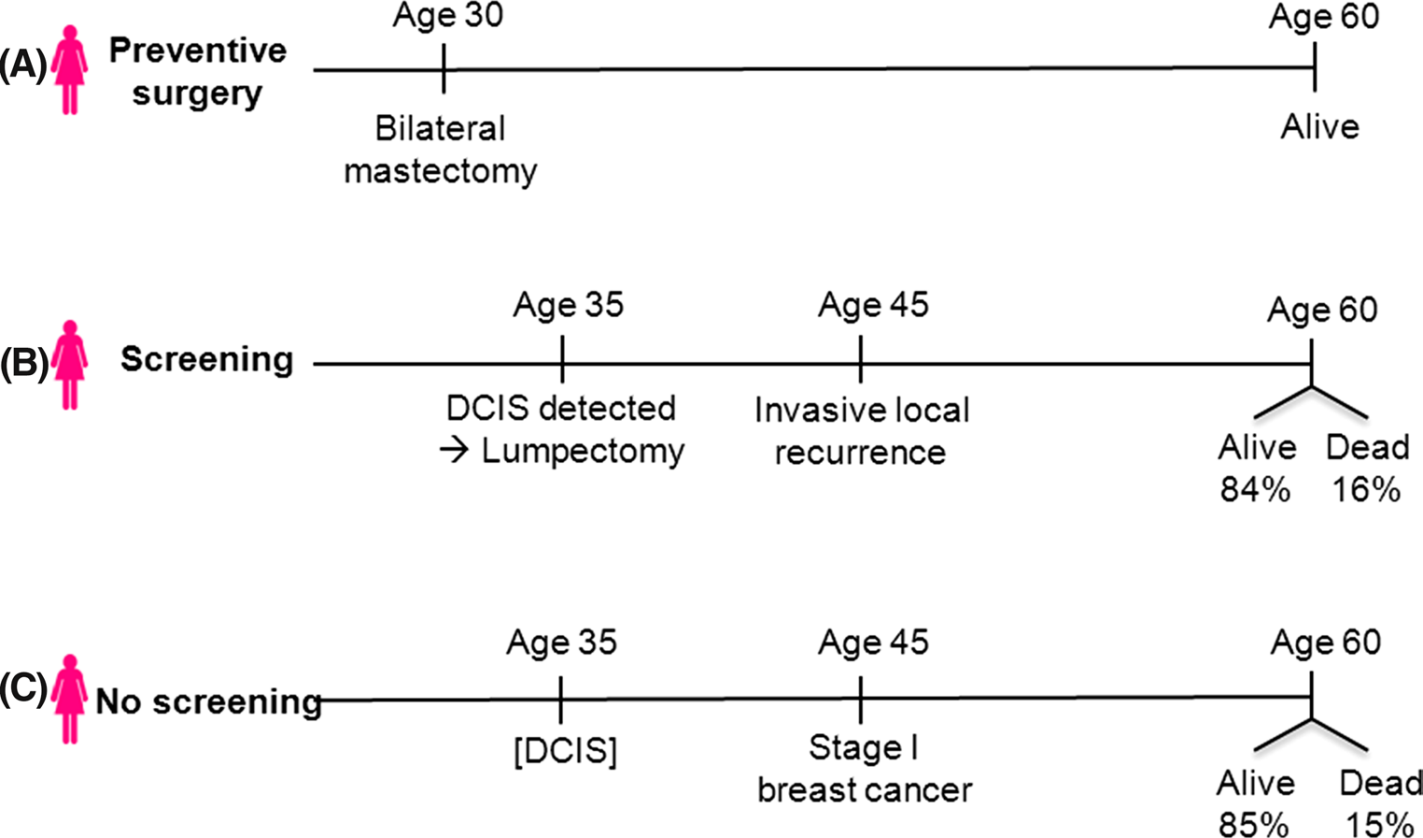
Tale of three women

Consider three women, born in the same year in the same city (Fig. 4).

*Woman A* is worried about getting breast cancer, and even though she has no family history or any dominant risk factor, she decides to have a bilateral prophylactic mastectomy at age 30. She is now age 60 and she is doing well with no cancer history. Unbeknownst to her, she was destined to develop DCIS at age 40, but this was pre-empted by the preventive surgery.

*Woman B* opts not for surgery, but goes for annual mammography. At age 40, she has an abnormal mammogram and a small focus of DCIS is discovered upon biopsy. She has breast-conserving surgery. The margins are clear. Despite this, 5 years later, she is diagnosed with an invasive recurrence in her breast, 2 cm in diameter. The nodes are clear.

*Woman C* does not go for mammography but instead goes to her doctor for an annual physical exam. At age 45, the physician feels a lump and the biopsy reveals a stage I invasive breast cancer, 2 cm in size. Nodes are clear.

**Fig. 5:**
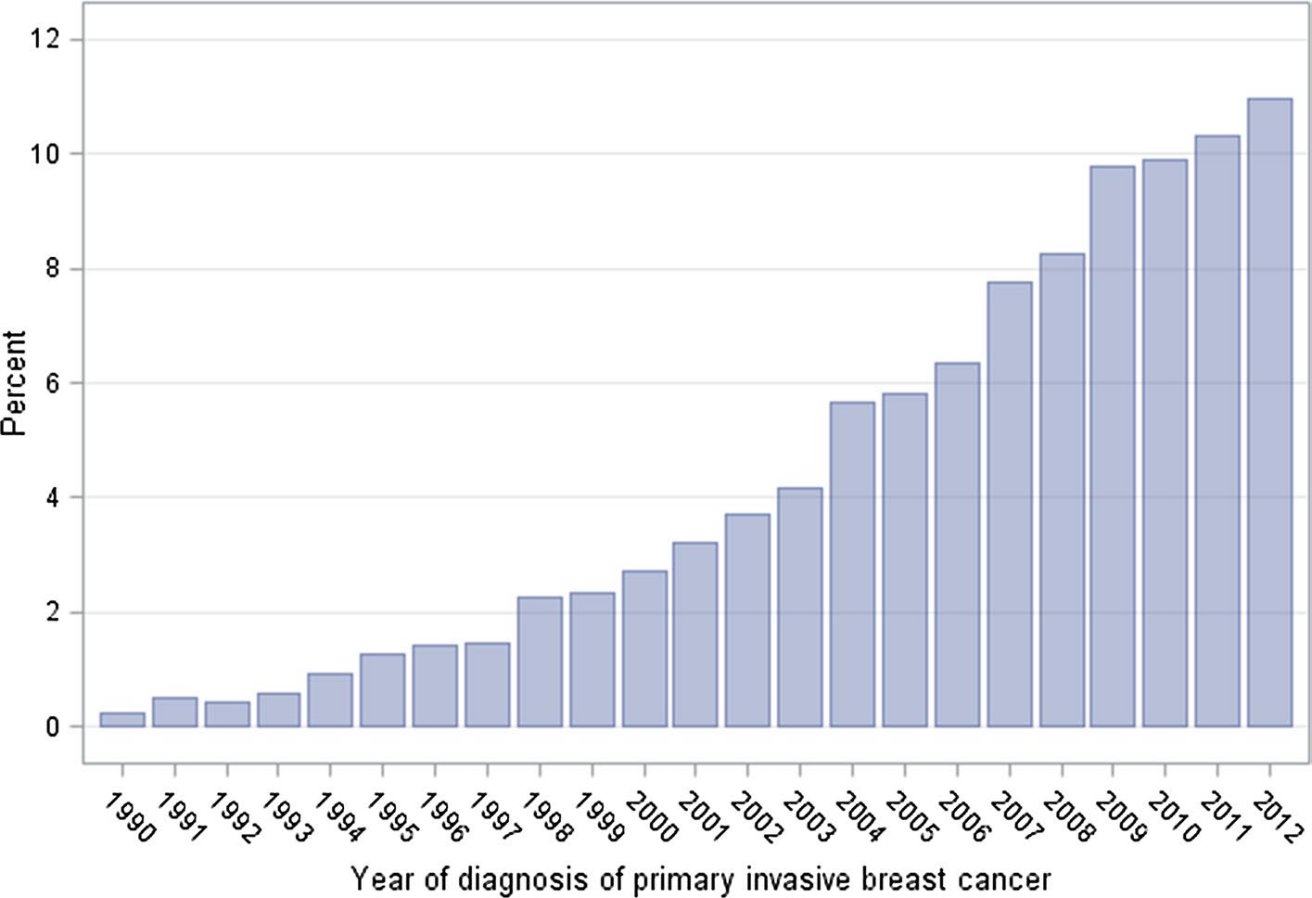
Proportion of cases of invasive breast cancer with a prior diagnosis of DCIS, by year of diagnosis of invasive breast cancer (SEER database)

In their medical synopses, woman B is described as having DCIS with an invasive recurrence and woman C is described as having primary invasive breast cancer. What we neglect to mention in this *gedankenexperiment* is that these three are the same woman. Before mammographic screening was introduced, women typically presented with invasive breast cancer and if they passed through an earlier stage of DCIS, no one was the wiser. After mammography was introduced, the incidence of DCIS rose by 500% (between 1983 and 2003) [50]. If we assume the underlying incidence of DCIS over time was stable, then the increase was the consequence of more extensive screening. The proportion of cases of invasive breast cancer with a prior record of DCIS grew steadily from 1980 to 2012 (Fig. 5) [51]. A woman who is diagnosed with DCIS and an ipsilateral invasive recurrence nowadays would likely have been diagnosed with primary invasive breast cancer in 1970.

In this light, we propose that in-breast invasive recurrences post-DCIS and primary invasive breast cancers are two facets of the same condition which differ according to the frame of reference of the observer. From the starting point of DCIS, risk factors which lead from progression of DCIS to invasive cancer include young age, high grade, and positive surgical margins [52]. From the point of view of the invasive cancer, the most important factor which predicts a prior diagnosis of DCIS is calendar year [53]. If we surmise correctly, breast cancers with and without a documented prior history of DCIS should carry a similar prognosis. Based on data from the Henrietta Banting Database at Women’s College Hospital in Toronto [54], we estimate the 15-year breast cancer-specific mortality rate after an invasive local recurrence (node-negative) following a diagnosis of DCIS to be 16% (Woman B). Based on data from the SEER registry [51], we estimate the 15-year breast cancer-specific mortality rate after a diagnosis of node-negative primary invasive breast cancer under 5 cm to be 15% (Woman C). The prognoses of Woman B and C are almost identical.

**Fig. 6:**
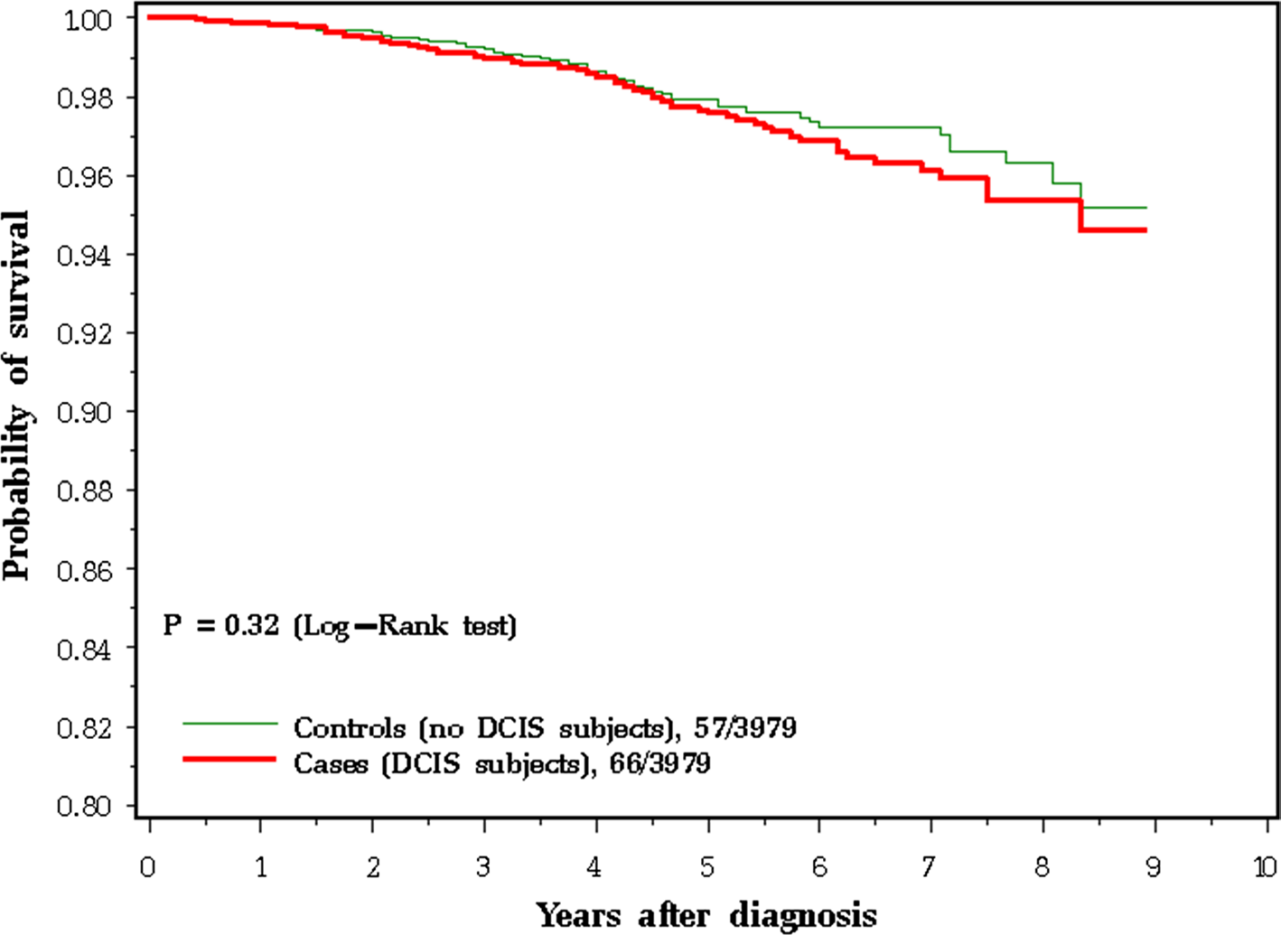
Breast cancer-specific survival at 9 years for 3979 matched pairs of patients with invasive breast cancer and a prior history of DCIS (cases) or no prior history of DCIS (controls)

It is also relevant to ask if a past history of DCIS is important in predicting the prognosis of a woman who presents with an invasive breast cancer. To this end, we analysed data from the SEER registry and compared the survival experience of women with invasive breast cancer, depending on whether or not they had a prior diagnosis of DCIS [54]. Women with invasive cancer, with and without prior DCIS, were matched on year of birth, age of diagnosis, size, nodal status, ER status, and type of surgery. In this analysis of 3979 matched pairs, a prior history of ipsilateral DCIS *did not* have an impact on prognosis (HR 0.91 for cancer-specific mortality) [54]. The clinical course of an invasive recurrence following DCIS was the same as that of a primary breast cancer (Fig. 6). In this respect, the invasive recurrence following DCIS also resembles a primary breast cancer. However, we also noted that a short time period from DCIS to invasive recurrence was a very strong risk factor for prognosis from the time of recurrence (i.e. less than 5 years versus more than 5 years). The previous studies have shown that a short time from primary invasive cancer to local invasive recurrence is a strong prognostic factor for death after local invasive recurrence [55, 56]. In this respect, the invasive recurrence following DCIS also resembles a local recurrence post-invasive cancer. If the invasive cancer following DCIS was a de novo primary cancer (rather than a recurrence of DCIS), then we would not expect the time from DCIS to invasive cancer to be predictive of prognosis.

Given these equivalencies, we conclude that primary invasive breast cancers and local invasive recurrences so closely resemble each other that they are likely to be two facets of the same condition—our tendency to separate them into two distinct clinical entities is based on historical grounds that should be revisited. We cannot distinguish a primary invasive cancer from a local recurrence on histologic examination, and to our knowledge, there are no molecular tests or gene signatures that allow a pathologist to do so.

## Does breast cancer metastasize?

In Part 1 above, we re-iterate the (accepted) position that a local recurrence following a case of invasive breast cancer is a marker of cancer aggressiveness (similar to positive lymph nodes) and is not a conduit of cancer spread [7]. From this, it follows logically that local recurrences do not metastasize.

In Part 2 above, we make the case that a local invasive recurrence following DCIS is similar in many respects to a local recurrence following invasive cancer and these do not have the ability to metastasize. We extend the argument that preventing local recurrence after an invasive primary cancer through mastectomy does not prevent death, and therefore, local invasive recurrences—after DCIS or after invasive cancer—do not have the ability to metastasize. Holzel et al. came to a similar conclusion about local recurrences in 2011 by examining data from the Munich Cancer Registry [57]. Interestingly, they thought that the metastatic potential of local recurrences was different from that of primary tumours (which did have the ability to metastasize) [57, 58].

**Fig. 7:**
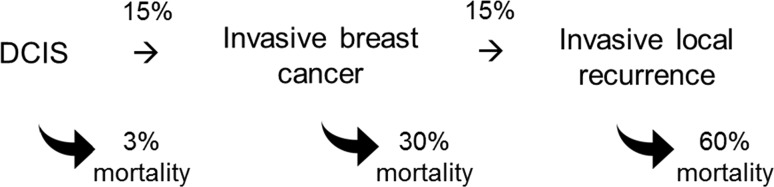
Hypothetical patient who presents with DCS has a lumpectomy followed by a local invasive cancer, a second conservative surgery, followed by a local invasive recurrence. The probability of going from one clinical state to the next is indicated by the upper arrows. The probability that the patient will die of breast cancer and indicated by the lower arrows

In Fig. 7, below, we illustrate a clinical course of a hypothetical patient who first experiences DCIS, then (after lumpectomy) has an invasive recurrence. She is treated with a mastectomy at the time of the recurrence. The probability of her experiencing a recurrence after the DCIS is roughly 15%, as is the probability of experiencing a (second) recurrence after the invasive cancer [54]. At each successive stage, the likelihood of her dying of breast cancer increases dramatically (from 3% to 30% to 60%) [51]; however, as discussed above, preventing progression from DCIS to invasive cancer or progression from invasive cancer to recurrent in-breast cancer through surgery does not reduce the possibility of death.

In Part 4 above, we argue that local invasive recurrences following a diagnosis of DCIS and primary de novo invasive breast cancers are variant representations of the same malignancy, and our categorical assignment depends on the frame of reference of the observer. This leads us to the surprising conclusion that what we now describe as primary invasive breast cancers do not have the ability to metastasize. In technical terms, the bulk of the cells that make up the tumour mass of a primary invasive breast cancer does not have the intrinsic ability to travel to distant organs and to re-initiate growth at a secondary site.

Here, our position deviates from the conventional view that a breast cancer, as we know it, is a fully formed malignancy, with innate abilities to grow and to metastasize, but we are able to support our position by reference to several clinical observations. These observations have, in common, the theme that some proportion of breast cancer cells can be left in situ after standard surgical treatment without adversely affecting the prognosis of the patient (Box C: Assumptions underlying parallel model in Appendix).

Fisher and Veronesi taught us that, in terms of mortality, patients treated with lumpectomy and mastectomy have similar prognoses [8, 9]. Nevertheless, a proportion of cancers removed by lumpectomy have positive margins; if the cancer has been cut through there will be residual cancer cells in the breast. Although positive margins increase the risk of local recurrence, it has not been shown that positive margins increase mortality [59, 60]. Some surgeons will argue that those cancer cells that are left behind are removed by re-excision. However, it has not been shown that in terms of mortality women who have cancers with positive margins do better in terms of death if they undergo re-excision [60]. The rate of local recurrence drops by 50%, but mortality drops by only a few percentage points [11]. Some medical oncologists will argue that adjuvant chemotherapy eliminates the remaining cells. However, the prognosis of women with lumpectomy versus mastectomy is similar if we exclude women who have had chemotherapy [8, 9].

Most radiotherapists will argue that adjuvant radiotherapy eliminates the few cancer cells remaining after lumpectomy and can prevent local invasive recurrence in the presence of positive margins. It has been shown in the meta-analysis of breast cancer randomized trials that, among women with invasive breast cancer treated with lumpectomy, that there is a small reduction in breast cancer mortality at 15 years associated with radiotherapy [11]. It is often stated by radiotherapists that for every four recurrences avoided, there would be one death averted. A recent EBCTG study compiles both locoregional and distant recurrences [11], but an earlier study by the same group (EBCTCG) considered only local recurrences [61]. In both studies, the proportional reduction of breast cancer mortality was small (about 15%) [11, 61]. The data in Table 2 summarize several studies that report both local recurrences and deaths [61]—note, however, that the rates of recurrence and mortality are grossly divergent and do not complement each other. Furthermore, after radiotherapy, there are far more deaths from breast cancer than there are local recurrences, i.e. we have prevented many recurrences with radiotherapy but few deaths. The reduction in mortality associated with radiation was greater for node-positive patients than node-negative patients, suggesting that the radiotherapy did not affect mortality through local control. We could just as easily conclude from looking at the aggregate data set that that local recurrence and mortality are two disjoint processes.

**Table 2:** Impact of radiotherapy after breast-conserving surgery on the rates of local recurrence and breast cancer mortality in the 2005 Early Breast Cancer Trialists’ Overview (EBCTG) report

	Node-negative patients		Node-positive patients	
	No radiotherapy	Radiotherapy	No radiotherapy	Radiotherapy
10-year risk of local recurrence (%)	29.2	10.0	46.5	13.1
10-year risk of breast cancer mortality (%)	20.3	17.4	45.2	36.5
Ratio deaths/recurrences	0.70	1.74	0.97	2.79

Magnetic resonance imaging (MRI) is now used as an adjunct to diagnostic staging for breast cancer patients. When MRI was added to classical staging techniques, 20% or more of women were found to have cancers that were more extensive than what was apparent using the traditional staging techniques [62]. When the more extensive cancers (multi-focal and multi-centric) were revealed by MRI, they were removed, often with mastectomy [63]. The proportion of women who had a mastectomy instead of a lumpectomy increased when MRI was employed; in the COMICE trial (Comparative Effectiveness of MRI in Breast Cancer), which was limited to conservation candidates, the conversion rate from lumpectomy to mastectomy was 7% for women who had an MRI versus 1% for women who did not have an MRI [64]. In other words, in the absence of MRI, about 20% of women who are treated with lumpectomy based on the traditional staging techniques, have residual cancer cells, but are unaware [62]. Studies to date (mostly small and with limited follow-up) have failed to show that adding MRI to the staging improves patient survival [65, 66]. That is, even though MRI is effective at detecting with accuracy the extent of a breast cancer, it is expensive and its use results in more extensive surgery, but does not reduce mortality and MRI is not endorsed in current surgical guidelines as a part of the workup of a patient with early stage breast cancer. It is possible that studies to date are too small and that the follow-up period is too short to show a benefit associated with MRI staging. It has been argued that the cancer cells that are left behind are not “clinically important”, as opposed to the ones that are surgically removed [67]. The argument that some cancer cells are clinically important and others are not is not based on any observable or measurable difference in the two cell populations—there is no way of telling them apart. It is possible that ‘clinical importance’ is determined by a critical number of cells, that is, the cells that are left behind are too few to be relevant. If so, this would be a departure from what medical oncologists consider a fundamental principal; i.e. that if untreated, cancer cells have the ability to flourish and will proliferate continuously until they threaten life.

## Tumour size as a marker of metastatic potential: mammography screening

Tumour size (diameter in centimeters) is a strong and consistent predictor of breast cancer survival [68]. That is, the size attained by the breast cancer at the time of diagnosis is a good predictor of whether or not the cancer has metastasized prior to diagnosis. From this, it has been inferred that the more cancer cells there are in the breast, the greater the probability that a cell or a small clone of cells will break off from the tumour mass and will lodge elsewhere and flourish. This conventional view (the sequential model) is based on the premise that cancer metastases originate from the in-breast cancer mass—the larger the cancer, the more cells there are and the greater the probability of a metastasis occurring. However, the size-survival association is also consistent with the alternate position that the in-breast tumour is a marker of cancer aggressiveness and not a source of metastases. In this sense, cancer within the breast is analogous to cancer within the lymph nodes; the greater the number of nodes involved, the higher the risk of recurrence.

The conventional view has also been challenged by Christoph Klein who notes that the mean time of cancer diagnosis to cancer recurrence is far too short to support a model that a metastasis is the result of a dislodging of a single cell and subsequent clonal exponential growth, and he has proposed an alternate model of parallel progression, whereby the primary (in-breast) cancer and the metastases arise simultaneously and grow synchronously [69]. The parallel model (versus the sequential model) is supported indirectly by the results of mammography screening trials. If the size of the cancer (in terms of the number of cancer cells) is proportional to its metastatic potential (and curability), then a 2 cm cancer, which contains eight times as many cells as a 1 cm cancer, should have a *much* worse prognosis than a 1 cm cancer, and reduction of cancer size from 2 to 1 cm through early detection should have a dramatic effect on mortality (perhaps as much as a 7/8th reduction) and not a mere drop of 30% or so (i.e. the best case scenario).

**Fig. 8:**
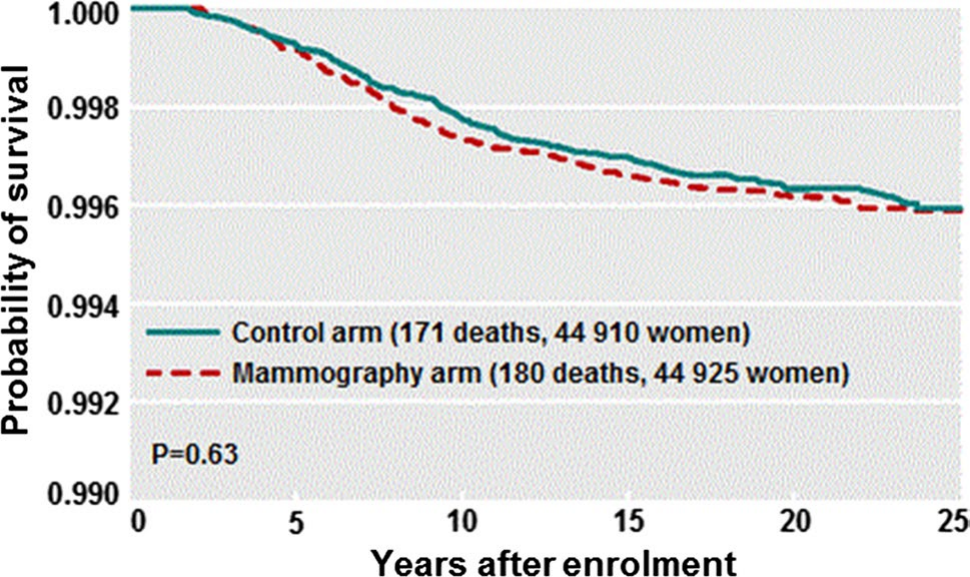
Breast cancer-specific survival of patients randomized to mammography or no mammography in the Canadian National Breast Screening Trial. 25-year follow-up

In our large Canadian randomized trial of breast cancer screening, we found no relationship between assignment to the mammography arm and subsequent mortality from breast cancer (HR 1.11) (Fig. 8) despite a significant drop in mean tumour size [70]. However, other breast cancer screening trials have reported a positive outcome (particularly the Swedish Two County Study [71]), and there is ongoing controversy regarding the value of mammographic screening [72] which we do not deal with in detail here. A reduction in mortality associated with mammography screening [71, 72] may be interpreted as evidence that (at least a subset of) breast cancers follow a sequential progression pattern (i.e. metastatic potential increases with tumour size). However, a mortality benefit from screening might also be achieved by earlier initiation of systemic therapies (chemotherapy and/or hormone therapy) and is also consistent with the parallel model. The latter interpretation is supported by the observation that a given decrease in tumour size is associated with a much larger mortality reduction for node-positive cancers (which are often treated with chemotherapy) than for node-negative cancers [73].

If the size of the breast cancer is a *marker of aggression* rather than a *source of metastases,* then the null outcome from mammography is to be expected (Fig. 8). Furthermore, we should expect the size of the mass to be of limited predictive ability and that the size/survival relationship is not constant, but varies according to pathologic subtype. In triple-negative breast cancer, in BRCA1-positive breast cancer, and in small HER2-positive breast cancers, the relationship between tumour size and survival is much attenuated, and for these women, tumour size is a very poor predictor of survival [74]. Furthermore, among breast cancers of 6 cm or greater, there is very little correlation between tumour size and node status or prognosis [75, 76]. For example, in a cross-sectional SEER-based study, the probability of lymph node metastases being present at diagnosis was 19% for 1 cm cancers, was 35% for 2 cm cancers, was 60% for 6 cm cancers, and was 65% for 10 cm cancers [75]. Assuming that large cancers were once small cancers, this implies that a breast cancer has 16% chance of metastasizing as it grows from 1 to 2 cm, but only a 5% chance of metastasizing as it grows from 6 to 10 cm (despite the fact that a 6 cm cancer contains approximately 200 times more cells). In contrast, the relationship between lymph node status and survival is canonical and varies little across clinical subtypes [77, 78]. Furthermore, it is often argued that there is a beneficial effect of screening, independent of tumour size, which is evidence in favour of screening [79, 80]. For example, we have found that tumour palpability is strong adverse prognostic factor in the Henrietta Banting database and we interpreted this to mean that palpability per se is a marker of aggressivity, rather like tumour grade, vessel count and nodal status, and to some extent, this may explain the relatively good prognosis of mammogram-detected cancers [80]. If the sequential model was true, we would expect screening to be highly effective. Furthermore, we would expect that the goal of screening would be realized largely by identifying cancers when they are small and node-negative. However, the negative statistical association between tumour size and survival is equally strong for node-positive breast cancer patients as it is for node-negative breast cancer patients [68, 73] and tumour size is predictive of survival even among women who present with stage IV breast cancers [81]. We interpret this to mean that tumour size, nodal status, and the presence of distant metastases are three complementary prognostic factors, as befits a parallel model, rather than a sequential model. It is also possible that under the parallel model, that screening has a beneficial effect, e.g. perhaps, smaller cancers are more likely to have fewer distant micrometastases than larger cancer, and therefore, these are more amenable to chemotherapy than larger cancers (with more micrometastases).

In a recent study in the New England Journal of Medicine [82], among all invasive breast cancers diagnosed from 2001 to 2013 in the SEER database, there was a dramatic difference in the distribution of biologic category according to tumour size; tumours with favourable biologic characteristics made up 38% of the tumours that were 1 cm or less in size, and this steadily decreased to only 9% of the tumours greater than 5 cm, whereas tumours with unfavourable biologic features made up only 14% of the tumours 1 cm or less, increasing to 36% of the tumours greater than 5 cm. Furthermore, while both tumour size and biologic features had a major influence on prognosis, large tumours with favourable biologic features had a better prognosis than small tumours with unfavourable biologic features. These observations suggest that tumour size may be a marker of tumour aggressivity, as are grade and ER status, rather than an indicator of the pool of cancer cells that are the source of dissemination.

Our model suggests that positive lymph nodes, in-breast lesions, and distant metastases result from dissemination via parallel pathways which are downstream from a common (but unseen) source. The malignant potential of the underlying cancer source predicts the size of the cancer, the number of nodes involved, and the presence of metastases at the time of diagnosis and thereafter.

By extension, it is disingenuous to refer to cancer stage to distinguish between patients with different prognoses, because the term ‘stage’ infers a natural course of progression, i.e. the patient passes through various ‘stages’ of cancer. If we consider the involvement of regional nodes and distant metastases to be independent and parallel processes, then the description should be cross-sectional and should not assume natural history, e.g. the size of the cancer, the nodal status, and the metastatic status should be stated explicitly using three separate variables.

In an early study, Horak et al. correlated the density of small blood vessels within the breast cancer with the probability of concurrent lymph node metastases [83]. In this study, microvessel density was strongly correlated with the presence of nodal metastases, such that only 2 of 50 tumours with vessel counts of 99/mm^3^ or less were node-positive, whereas 32 of 39 tumours with counts above 140/mm^2^ were node-positive. From this, the authors infer that the extent of angiogenesis within the primary cancer is an indicator of its metastatic potential. This interpretation has appeal, but the data are also consistent with the model that the breast primary and the node metastasis both derive from a common precursor. Similarly, the observation that a breast primary cancer and the distant metastases are concordant for one or more genetic markers is no guarantee that the metastasis is a subclone of the breast cancer—it might be that the two have a common ancestor. Indeed, Hosseini et al. [84], in a recent paper, propose that the latter scenario is, in fact, the case—a comparison of the early cancer precursor, the in-breast primary, and the metastases found that the metastatic lesion more closely resembled the (hypothetical) precursor lesion than the much larger breast tumour.

**Fig. 9:**
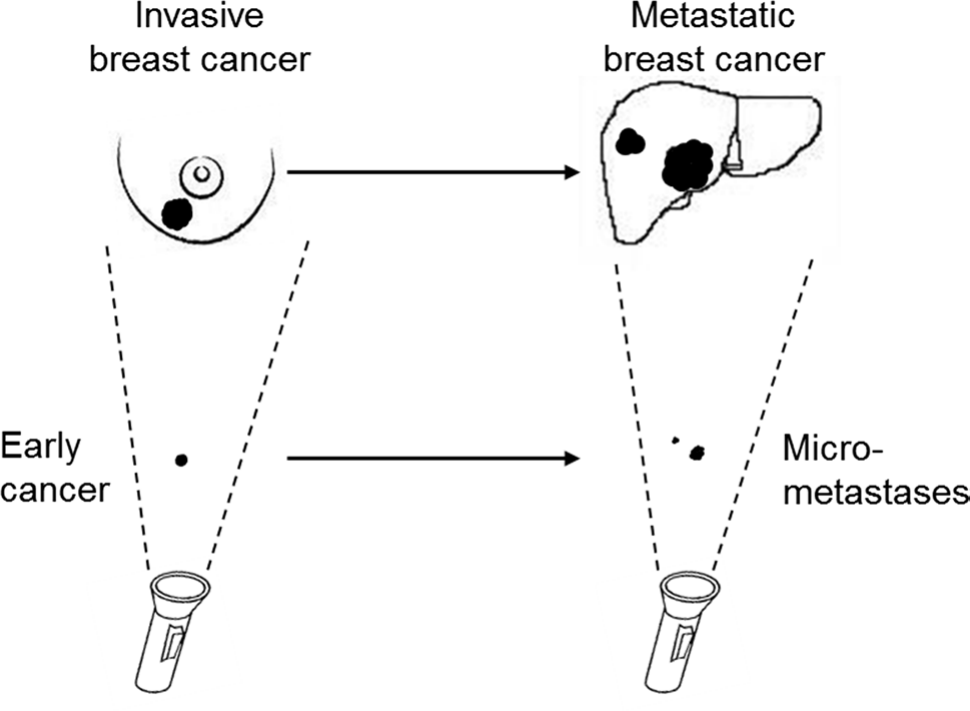
‘Shadows on the Wall‘. Our version of causality is influenced by the visible manifestations of cancer at various times. We observe a breast mass followed by signs of metastatic disease several months/years later (upper path). These observable events are gross manifestations of subtle events which occur earlier but are not detected by the conventional imaging technologies (lower path)

We believe that the conventional view of the natural history of breast cancer is based on questionable assumptions which are derived from an incorrect interpretation of a number of clinical observations. Given the typical timeline of the clinical manifestations of breast cancer (an in-breast mass with or without positive nodes, followed by metastatic disease), it was reasonable for the observer to conclude that the process was sequential and the patient progressed through various stages. We propose here that the connections between the various manifestations of breast cancer (from DCIS to clinically apparent metastases) should not be inferred from what is visualized by the pathologist (‘the shadows on the wall’) but rather from the clinical data on patterns of recurrence and mortality seen through the epidemiologist’s lens. This is represented in Fig. 9.

The critical events occur unseen; normal cells are transformed into small clusters of cancer cells with multi-potent metastatic potential and these give rise to early lesions in the breast as well as subclinical latent metastatic deposits. Typically, months to years will pass before the metastases become manifest, and during this time period, we may see the emergence of breast cancer in the breast and in the nodes. The relative timing of the appearance of cancer cells in the breast in the nodes and in distant organs is governed by factors which determine the dynamic growth of cancer within the various niches. It is of interest in this regard that the majority of cancer patients who develop distant metastases present with cancer stages I to III (i.e. they rarely present at stage IV) [51], whereas the majority of women who have positive axillary lymph nodes and breast cancer present with cancer in both sites synchronously (i.e. lymph node status rarely transitions from negative to positive after the initial diagnosis) [85, 86]. These observations suggest that the early cancer-forming cells have many properties in common with cancer stem cells and cancer progenitor cells.

## Implications for prevention

The parallel model proposed here may be relevant to cancer prevention. Under the conventional (sequential) model, we expect a reduction in cancer mortality to parallel a reduction in cancer incidence and prevention trials were designed accordingly, with incidence as the primary endpoint. However, as outlined in Fig. 2, we speculate that the pathways from early cancers to in breast tumours and to metastases are distinct and separable, and that there is no pathway from in-breast cancer to metastases. Under the parallel model, a factor that prevents in-breast cancer (i.e. incidence) may not prevent cancer deaths. We have recently summarized the epidemiologic evidence that tamoxifen chemoprevention prevents cancer but does not appear to prevent death [87]. This may be because tamoxifen interrupts one pathway (the in-breast recurrence pathway) but does not interrupt the other (the metastatic pathway). This could also be true for lifestyle interventions, such as diet and exercise, and, therefore, to evaluate the effectiveness of a prevention intervention, it is necessary to consider breast cancer mortality as the primary endpoint.

## Implications for screening

The strong association between cancer size and mortality is not under dispute, but under the parallel model, we no longer support the paradigm of early detection, namely that if we can identify a cancer when it is small we can advance the date of diagnosis and this in turn will reduce the likelihood of subsequent mortality. Under the parallel model, tumour size is a marker of aggressiveness, as is lymph node status and tumour palpability, but the tumour is not a conduit of metastatic cells.

### Implications for treatment

Under the sequential model, if we can identify a cancer when it is small and before it has metastasized we expect to cure it through surgery alone. If the metastases are local (regional lymph nodes), the chance of curing the patients with surgery and chemotherapy is high and a proportion of cancers with subclinical latent metastases will also be cured by a combination of chemotherapy and surgery.

In the parallel model, the potential for metastases is present from the outset—not once the cancer reaches a particular size. Chemotherapy should be introduced early in the clinical course. We should have as a research goal identifying cancers which have a high chance of metastases when small and to identify metastatic disease before it is clinically apparent by new sensitive imaging and molecular techniques, possibly including circulating tumour DNA.

The goal of preventing local invasive cancer after DCIS is a laudable one, but one that has limited clinical benefit in that this will not prevent mortality. If we wish to reduce the mortality rate for women with DCIS, it is necessary to have the means to identify women at sufficiently high risk to be candidates for systemic therapy.

## Conclusions

Our understanding of the nature of breast cancer is formulated by knowledge collected and synthesized over several decades. Traditionally, we see each breast cancer at a moment in time and within a narrow visual frame, rather than as a dynamic process, whereby cancer cells appear (and possibly disappear) within the breast and elsewhere. The conventional model describes breast cancers as a mass of cells, malignant in appearance, and which have the ability to divide and to metastasize and typically which pass through four stages before death ensues. We have reviewed here many of the seminal clinical findings over the past century and have tried to reconcile some contradictions and explain some outliers. By close analysis of patient outcomes, we challenge the conventional view of breast cancer progression as a sequential series of events. We believe that the temporal sequence of invasion, growth, and metastasis should be reinterpreted according to a new frame of reference (summarized in Box C: Assumptions underlying parallel model in Appendix). If we accept these assumptions, then it follows that minute cancers are capable of metastasizing prior to invasion into the breast and in contrast, what we currently consider to be malignant breast cancers are not the source of metastases.

## Appendix

### Box A: Historical timeline of breast cancer

1894: First published results from patients treated with the Halsted radical mastectomy [3]
1972: First guidelines published for the use of radiotherapy in treatment of breast cancer [88]
1975: Combination chemotherapy shown to be effective as adjuvant treatment in breast cancer [89]
1980: First guidelines published for the use of mammography in early detection of breast cancer [90]
1981: First published results from Veronesi’s randomized trial of mastectomy vs. lumpectomy [91]
1983: Mammogram-detected intra-ductal breast cancer described as Ductal Carcinoma In Situ [92]
1985: First published results from Fisher’s randomized trial of mastectomy vs. lumpectomy [93]

### Box B: The essential paradoxes

- DCIS is Stage 0 breast cancer. 2% of cases of DCIS are node-positive. Node-positive breast cancers are Stage 2. Stage 2 breast cancers are not DCIS.
- Primary breast cancers and invasive recurrences are not distinguishable either in terms of histology or prognosis. Their classification depends on context.
- The paradox of screening: reduction in size at diagnosis does not imply reduction in mortality.
- 0.3% of breast cancers present with metastases but no primary cancer
- Retention of cancer cells after surgery does not impair survival.

### Box C: Assumptions underlying parallel model

- The prevention of local recurrence following DCIS does not prevent death from breast cancer.
- The prevention of local recurrence following primary invasive breast cancer does not prevent death from breast cancer.
- The prognosis of women with invasive breast cancer is not impacted by a prior history of DCIS.
- Cancer size is a marker of breast cancer aggressiveness and not a conduit of cancer spread.
- In-breast invasive cancers do not metastasize.

### Box D: Implications of parallel model

- Tumour stage should be replaced by size, nodes, and metastases (three separate variables).
- Early detection does not save lives.
- Preventing cancer does not guarantee preventing death from cancer.
- Chemotherapy should be introduced early.

## Footnotes

1 Consider for example the observation that men with nicotine-stained teeth have a much higher risk of dying of lung cancer than men with unstained teethed (perhaps 20-fold higher). This does not imply that brushing your teeth prevents lung cancer.

## References

[1] Fisher B, Redmond C, Poisson R (1989). Eight-year results of a randomized clinical trial comparing total mastectomy and lumpectomy with or without irradiation in the treatment of breast cancer. N Engl J Med.

[2] Veronesi U, Volterrani F, Luini A (1990). Quadrantectomy versus lumpectomy for small size breast cancer. Eur J Cancer.

[3] Halsted WS (1984). The Results of Operations for the Cure of Cancer of the Breast Performed at the Johns Hopkins Hospital from June, 1889, to January, 1894. Ann Surg.

[4] NIH consensus conference (1991). Treatment of early-stage breast cancer. JAMA.

[5] Du X, Freeman DH, Syblik DA (2000). What drove changes in the use of breast conserving surgery since the early 1980s? The role of the clinical trial, celebrity action and an NIH consensus statement. Breast Cancer Res Treat.

[6] Foote RL, Johnson RE, Donohue JH (2008). Trends in surgical treatment of breast cancer at Mayo Clinic 1980-2004. Breast.

[7] Fisher B, Anderson S, Fisher ER (1991). Significance of ipsilateral breast tumour recurrence after lumpectomy. Lancet.

[8] Fisher B, Anderson S, Bryant J (2002). Twenty-year follow-up of a randomized trial comparing total mastectomy, lumpectomy, and lumpectomy plus irradiation for the treatment of invasive breast cancer. N Engl J Med.

[9] Veronesi U, Cascinelli N, Mariani L (2002). Twenty-year follow-up of a randomized study comparing breast-conserving surgery with radical mastectomy for early breast cancer. N Engl J Med.

[10] Killander F, Karlsson P, Anderson H (2016). No breast cancer subgroup can be spared postoperative radiotherapy after breast-conserving surgery. Fifteen-year results from the Swedish Breast Cancer Group randomized trial, SweBCG 91 RT. Eur J Cancer.

[11] Darby S, McGale P, Early Breast Cancer Trialists’ Collaborative Group (EBCTCG) (2011). Effect of radiotherapy after breast-conserving surgery on 10-year recurrence and 15-year breast cancer death: meta-analysis of individual patient data for 10,801 women in 17 randomised trials. Lancet.

[12] Narod SA, Iqbal J, Giannakeas V, Sopik V, Sun P (2015). Breast cancer mortality after a diagnosis of ductal carcinoma in situ. JAMA Oncol.

[13] Tavassoli FA (1998). Ductal carcinoma in situ: introduction of the concept of ductal intraepithelial neoplasia. Mod Pathol.

[14] Boyages J (2014). DCIS of the breast: taking control.

[15] Wellings SR, Jensen HM, Marcum RG (1975). An atlas of subgross pathology of the human breast with special reference to possible precancerous lesions. J Natl Cancer Inst.

[16] Porter D, Lahti-Domenici J, Keshaviah A (2003). Molecular markers in ductal carcinoma in situ of the breast. Mol Cancer Res.

[17] Lampejo OT, Barnes DM, Smith P, Millis RR (1994). Evaluation of infiltrating ductal carcinomas with a DCIS component: correlation of the histologic type of the in situ component with grade of the infiltrating component. Semin Diagn Pathol.

[18] Lakhani SR (1999). The transition from hyperplasia to invasive carcinoma of the breast. J Pathol.

[19] Allred DC, Mohsin SK, Fuqua SAW (2001). Histological and biological evolution of human premalignant breast disease. Endocr Relat Cancer.

[20] Burstein HJ, Polyak K, Wong JS, Lester SC, Kaelin CM (2004). Ductal carcinoma in situ of the breast. N Engl J Med.

[21] Omer ZB, Hwang ES, Esserman LJ, Howe R, Ozanne EM (2013). Impact of ductal carcinoma in situ terminology on patient treatment preferences. JAMA Intern Med.

[22] Early Breast Cancer Trialists’ Collaborative Group (EBCTG) Correa C, McGale P et al (2010) Overview of the randomized trials of radiotherapy in ductal carcinoma in situ of the breast. J Natl Cancer Inst Monogr 41:162–169

[23] Elshof LE, Schmidt MK, Rutgers EJ, van Leeuwen FE, Wesseling J, Schaapveld M (2017). Cause-specific mortality in a population-based cohort of 9799 women treated for ductal carcinoma in situ. Ann Surg.

[24] Wadsten C, Garmo H, Fredrikkson I, Sund M, Warnberg F (2017). Risk of death from breast cancer after treatment for ductal carcinoma in situ. Br J Surg.

[25] Wapnir IL, Dignam JJ, Fisher B (2011). Long-term outcomes of invasive ipsilateral breast tumor recurrences after lumpectomy in NSABP B-17 and B-24 randomized clinical trials for DCIS. J Natl Cancer Inst.

[26] Van Zee KJ, Barrio AV, Tchou J, Society of Surgical Oncology Breast Disease Site Work Group (2016). Treatment and long-term risks for patients with a diagnosis of ductal carcinoma in situ. JAMA Oncol.

[27] Sopik V, Sun P, Narod SA (2017). Impact of microinvasion on breast cancer mortality in women with ductal carcinoma in situ. Breast Cancer Res Treat.

[28] Fallowfield L, Francis A, Thompson AM (2016). Effects of standard treatments for ductal carcinoma in situ-making informed choices. JAMA Oncol.

[29] Sanger N, Engels K, Graf A (2014). Molecular markers as prognostic factors in DCIS and small invasive breast cancers. Geburtshilfe Frauenheilkd.

[30] Franken B, de Groot MR, Mastboom WJ (2012). Circulating tumor cells, disease recurrence and survival in newly diagnosed breast cancer. Breast Cancer Res.

[31] Sänger N, Effenberger KE, Riethdorf S (2011). Disseminated tumor cells in the bone marrow of patients with ductal carcinoma in situ. Int J Cancer.

[32] Hüsemann Y, Geigl JB, Schubert F (2008). Systemic spread is an early step in breast cancer. Cancer Cell.

[33] Gruber IV, Hartkopf AD, Hahn M (2016). Relationship between hematogenous tumor cell dissemination and cellular immunity in DCIS Patients. Anticancer Res.

[34] Banys M, Hahn M, Gruber I (2014). Detection and clinical relevance of hematogenous tumor cell dissemination in patients with ductal carcinoma in situ. Breast Cancer Res Treat.

[35] El Hage Chehade H, Headon H, Wazir U, Abtar H, Kasem A, Mokbel K (2017). Is sentinel lymph node biopsy indicated in patients with a diagnosis of ductal carcinoma in situ? A systematic literature review and meta-analysis. Am J Surg.

[36] Osako T, Iwase T, Kimura K, Horii R, Akiyama F (2013). Detection of occult invasion in ductal carcinoma in situ of the breast with sentinel node metastasis. Cancer Sci.

[37] Zetterlund L, Stemme S, Arnrup H, de Boniface J (2014). Incidence of and risk factors for sentinel lymph node metastasis in patients with a postoperative diagnosis of ductal carcinoma in situ. Br J Surg.

[38] Gadre SA, Perkins GH, Sahin AA, Sneige N, Deavers MT, Middleton LP (2008). Risk of needle tract seeding of breast cancer: cytological results derived from core wash material. Breast Cancer Res Treat.

[39] Shymala K, Girish HC, Murgod S (2014). Risk of tumor cell seeding through biopsy and aspiration cytology. J Int Soc Prev Community Dent.

[40] Hansen NM, Ye X, Grube BJ, Giuliano AE (2004). Manipulation of the primary breast tumor and the incidence of sentinel node metastases from invasive breast cancer. Arch Surg.

[41] Uematsu T, Kasami M (2008). Risk of needle tract seeding of breast cancer: cytological results derived from core wash material. Breast Cancer Res Treat.

[42] Liebens F, Carly B, Cusumano P (2009). Breast cancer seeding associated with core needle biopsies: a systematic review. Maturitas.

[43] Narod SA, Ahmed H, Sopik V (2017). Commentary: wherein the authors attempt to minimise the confusion generated by their study “Breast cancer mortality after a diagnosis of ductal carcinoma in situ” by several commentators who disagree with them and a few who don’t: a qualitative study. Curr Oncol.

[44] Cheatle GL (1906). Early recognition of cancer of the breast. BMJ.

[45] Fisher ER, Sass R, Fisher B, Wickerham L, Paik SM (1986). Pathologic findings from the National Surgical Adjuvant Breast Project (protocol 6). I. Intraductal carcinoma (DCIS). Cancer.

[46] Helvie MA, Chang JT, Hendrick RE, Banerjee M (2014). Reduction in late-stage breast cancer incidence in the mammography era: implications for overdiagnosis of invasive cancer. Cancer.

[47] Hofvind S, Lee CI, Elmore JG (2012). Stage-specific breast cancer incidence rates among participants and non-participants of a population-based mammographic screening program. Breast Cancer Res Treat.

[48] Bhathal PS, Brown RW, Lesueur GC, Russell IS (1985). Frequency of benign and malignant breast lesions in 207 consecutive autopsies in Australian women. Br J Cancer.

[49] Kuhl C, Bieling H, Strobel K, Leutner C, Schild H, Schrading S (2015) Breast MRI screening of women at average risk of breast cancer: an observational cohort study. J Clin Oncol 33:suppl 28S,abstr 1

[50] Kerlikowske K (2010). Epidemiology of ductal carcinoma in situ. J Natl Cancer Inst Monogr.

[51] Howlader N, Noone AM, Krapcho M et al. SEER Cancer Statistics Review, 1975–2012. National Cancer Institute, Bethesda. http://seer.cancer.gov/csr/1975_2012/, based on November 2014 SEER data submission, posted to the SEER web site, April 2015

[52] Narod SA, Rakovitch E (2014). A comparison of the risks of in-breast recurrence after a diagnosis of dcis or early invasive breast cancer. Curr Oncol.

[53] Sopik V, Iqbal J, Sun P, Narod SA (2016). Impact of a prior diagnosis of DCIS on survival from invasive breast cancer. Breast Cancer Res Treat.

[54] Sopik V, Nofech-Mozes S, Sun P, Narod SA (2016). The relationship between local recurrence and death in early-stage breast cancer. Breast Cancer Res Treat.

[55] Dent R, Valentini A, Hanna W (2014). Factors associated with breast cancer mortality after local recurrence. Curr Oncol.

[56] Gosset M, Hamy AS, Mallon P (2016). Prognostic impact of time to ipsilateral breast tumor recurrence after breast conserving surgery. PLoS ONE.

[57] Hölzel D, Emeny RT, Engel J (2011). True local recurrences do not metastasize. Cancer Metastasis Rev.

[58] Engel J, Eckel R, Kerr J (2003). The process of metastasisation for breast cancer. Eur J Cancer.

[59] Park CC, Mitsumori M, Nixon A (2000). Outcome at 8 years after breast-conserving surgery and radiation therapy for invasive breast cancer: influence of margin status and systemic therapy on local recurrence. J Clin Oncol.

[60] Bernardi S, Bertozzi S, Londero AP, Gentile G, Angione V, Petri R (2014). Influence of surgical margins on the outcome of breast cancer patients: a retrospective analysis. World J Surg.

[61] Clark M, Collins R, Darby S (2005). Effects of radiotherapy and of differences in the extent of surgery for early breast cancer on local recurrence and 15-year survival: an overview of the randomised trials. Lancet.

[62] Houssami N, Ciatto S, Macaskill P (2008). Accuracy and surgical impact of magnetic resonance imaging in breast cancer staging: systematic review and meta-analysis in detection of multifocal and multicentric cancer. J Clin Oncol.

[63] Neri A, Marrelli D, Megha T (2015). Clinical significance of multifocal and multicentric breast cancers and choice of surgical treatment: a retrospective study on a series of 1158 cases. BMC Surg.

[64] Turnbull L, Brown S, Harvey I (2010). Comparative effectiveness of MRI in breast cancer (COMICE) trial: a randomised controlled trial. Lancet.

[65] Solin LJ, Orel SG, Hwang WT, Harris EE, Schnall MD (2008). Relationship of breast magnetic resonance imaging to outcome after breast-conservation treatment with radiation for women with early-stage invasive breast carcinoma or ductal carcinoma in situ. J Clin Oncol.

[66] Sung JS, Li J, Da Costa G (2014). Preoperative breast MRI for early-stage breast cancer: effect on surgical and long-term outcomes. AJR Am J Roentgenol.

[67] Wood WC (2013). Close/positive margins after breast-conserving therapy: additional resection or no resection?. Breast.

[68] Saadatmand S, Bretveld R, Siesling S, Tilanus-Linthorst MM (2016). Influence of tumour stage at breast cancer detection on survival in modern times: population based study in 173,797 patients. BMJ.

[69] Klein CA (2009). Parallel progression of primary tumours and metastases. Nat Rev.

[70] Miller AB, Wall C, Baines CJ, Sun P, To T, Narod SA (2014). Twenty five year follow-up for breast cancer incidence and mortality of the Canadian National Breast Screening Study: randomised screening trial. BMJ.

[71] Tabar L (2011). Swedish two-county trial: impact of mammographic screening on breast cancer mortality during 3 decades. Radiology.

[72] Nelson HD, Cantor A, Humphrey L et al (2016) Screening for Breast Cancer: A Systematic Review to Update the 2009 U.S. Preventive Services Task Force Recommendation [Internet]. Rockville (MD): Agency for Healthcare Research and Quality 26889531

[73] Narod SA (2012). Tumour size predicts long-term survival among women with lymph node-positive breast cancer. Curr Oncol.

[74] Foulkes WD, Reis-Filho JS, Narod SA (2010). Tumor size and survival in breast cancer–a reappraisal. Nat Rev Clin Oncol.

[75] Ries LAG, Young JL, Keel GE, Eisner MP, Lin YD. Horner MJ (2007) SEER Survival Monograph: Cancer Survival Among Adults: U.S. SEER Program, 1988-2001, Patient and Tumor Characteristics. National Cancer Institute, SEER Program, NIH Pub. No. 07-6215, Bethesda, MD

[76] Yu K, Jiang YZ, Chen S (2012). Effect of large tumor size on cancer-specific mortality in node-negative breast cancer. Mayo Clin Proc.

[77] Dent R, Hanna WM, Trudeau M, Rawlinson E, Sun P, Narod SA (2009). Time to disease recurrence in basal-type breast cancers: effects of tumor size and lymph node status. Cancer.

[78] Zhang Y, Mo M, Li JW (2016). Better predictive value of axillary lymph node (ALN) status after systemic therapy for operable HER2-overexpressing breast cancer: a single-institution retrospective study. Eur J Surg Oncol.

[79] Mook S, Van ‘t Veer LJ, Rutgers EJ (2011). Independent prognostic value of screen detection in invasive breast cancer. J Natl Cancer Inst.

[80] Narod SA (2011). Age of diagnosis, tumor size, and survival after breast cancer: implications for mammographic screening. Breast Cancer Res Treat.

[81] Eng LG, Dawood S, Sopik V (2016). Ten-year survival in women with primary stage IV breast cancer. Breast Cancer Res Treat.

[82] Lannin DR, Wang S (2017). Are small breast cancers good because they are small or small because they are good?. New Engl J Med.

[83] Horak ER, Leek R, Klenk N (1992). Angiogenesis, assessed by platelet/endothelial cell adhesion molecule antibodies, as indicator of node metastases and survival in breast cancer. Lancet.

[84] Hosseini H, Obradović MM, Hoffmann M (2016). Early dissemination seeds metastasis in breast cancer. Nature.

[85] Vujovic O, Yu E, Cherian A, Dar AR, Stitt L, Perera F (2009). The number of axillary nodes removed as a predictor of regional recurrence in node negative breast cancer. Radiother Oncol.

[86] Linnaus ME, Dueck AC, Kosiorek HE (2015). Regional recurrence in the era of sentinel lymph node biopsy. Am J Surg.

[87] Sopik V, Narod SA (2015). Overdiagnosis in breast cancer chemoprevention trials. Curr Oncol.

[88] Montague ED (1972). Proceedings: radiotherapy of breast cancer: guidelines according to features of disease. Proc Natl Cancer Conf.

[89] Bonadonna G, Brusamolino E, Valagussa P (1976). Combination chemotherapy as an adjuvant treatment in operable breast cancer. N Engl J Med.

[90] American Cancer Society (1980). Guidelines for the cancer-related checkup: recommendations and rationale. CA Cancer J Clin.

[91] Veronesi U, Saccozzi R, Del Vecchio M (1981). Comparing radical mastectomy with quadrantectomy, axillary dissection, and radiotherapy in patients with small cancers of the breast. N Engl J Med.

[92] Holland R, Hendriks JH, Mravunac M (1983). Mammographically occult breast cancer. A pathologic and radiologic study. Cancer.

[93] Fisher B, Bauer M, Margolese R (1985). Five-year results of a randomized clinical trial comparing total mastectomy and segmental mastectomy with or without radiation in the treatment of breast cancer. N Engl J Med.

